# 

**Published:** 1860-04

**Authors:** 


### CONTENTS FOR APRIL, 1860.

### ORIGINAL COMMUNICATIONS.

An account of an Epidemic of Diphtheria. By W. L. WELLS, M. D., of Milwaukee, .. 193
Surgical Notes. By E. ANDREWS, M. D .......... 218
Cases and Reflections on the Use of the Gum Elastic Air Bag. By W. H. Byford, M. D. 220
Mutilation of a Child by a Homoeopathic Physician. By HIRAM NANCE, M. D........ 222
Clinical Reports. By N. S. DAVIS, M. D.......... 225
Chicago Academy of Medical Sciences.......... 237

### BOOK AND PAMPHLET NOTICES.

A Guide to the Practical Study of Diseases of the Eye; with an outline of their Medical and Operative Treatment. By JAMES DIXON, F. R C. S.......... 240
A Practical Treatise on Fractures and Dislocations. By FRANK HASTINGS HAMILTON, M. D., Professor of Surgery in the University of Buffalo, etc.,.......... 240
Clinical Lectures on Certain Acute Diseases. By ROBERT BENTLEY TODD, M. D., F. R. S., Author of Lectures on diseases of the Urinary Organs, etc.......... 240

### SELECTIONS.

Goitre successfully treated by large doses of Bromide of Potassium and Liquor Potassoe 247
Turpentine in Hæmoptysis.......... 248
Hernia Tes is from Strumous Deposit.......... 249
Elevation of the Subclavian Artery above the Clavicle, in a Phthisical Patient....... 250
Enchondroma of the big toe for twenty years.......... 251
Severe Burn of the Neck, treated by extension from the beginning.......... 252
Elephantiasis of the Clitoris,.......... 252

### EDITORIAL.

First Annual Commencement of Medical Department of Lind Uniuersity.......... 253

Illinois State Medical Society,.......... 256

Delegates to the State Society.......... 256

